# Copy of a Letter from Dr. Marshall to Mr. Ring, Dated Gibraltar, August 23, 1800

**Published:** 1800-11

**Authors:** J. H. Marshall


*To the Editors of the Medical and Phyſical Journal.*

GENTLEMEN,

PLEASE to inſert the incloſed copy of Dr. Marſhall's letter in the next Number of your Journal. I am,

GENTLEMEN,

Your's, &c.

JOHN RING.

*New-Street, Hanover-Square,*

*October* 16, 1800.

---

*Copy of a Letter from Dr. Marſhall to Mr. Ring, dated Gibraltar, Auguſt* 23, 1800.

MY DEAR SIR,

I make no doubt of the ſatisfaction you will feel when I inform you of the very polite reception and great attention we have met with from the Governor-General O'Hara, who intereſts himſelf much in the ſucceſs of that great diſcovery of which which we are the miſſionaries; and ſet the example to the garriſon by having his own infant inoculated. We have ſince inoculated the ſoldiers of the garriſon and their children, who have not had the Small-pox, and to-morrow we expect to ſail for Minorca, with recommendations to inoculate the Engliſh army now lying there.

From the medical men here, we have met with the moſt liberal and polite attention; and I am further happy to add, that all are equally convinced of the efficacy of the Cow-pox in reſiſting the Small-pox, and of the great reward due to our friend, Dr. Jenner, for the benefit he has conferred upon ſociety and the world at large, by his inveſtigation of this ſo peculiarly mild and ſafe diſeaſe.

In this warm, and in compariſon with England, hot climate, we have not obſerved any diſſimilarity of ſymptoms in the progreſs of the diſeaſe from what is uſual in England.

The Governor has applied to the court of Madrid, to obtain liberty for us to go there to inoculate; and it is probable, that upon our return to England, we may ſtop there a ſhort time. Some of the matter we uſed for inoculation here, was what you obligingly furniſhed me with; and we find it perfectly efficacious, although no precaution had been uſed as to the preſerving it more than putting it into a ſmall phial.

I ſhall from Minorca ſend you the reſult of our inoculation, though I have no doubt of its proving as ſucceſsful as it has done here.

Dr. Walker begs to preſent with me our joint reſpects and good wiſhes. Believe me ever

Your obliged friend,

J. H. MARSHALL.

